# Oligonucleotides and ND-FISH Displaying Different Arrangements of Tandem Repeats and Identification of *Dasypyrum villosum* Chromosomes in Wheat Backgrounds

**DOI:** 10.3390/molecules22060973

**Published:** 2017-06-14

**Authors:** Zhiqiang Xiao, Shuyao Tang, Ling Qiu, Zongxiang Tang, Shulan Fu

**Affiliations:** 1 Provincial Key Laboratory of Plant Breeding and Genetics, Sichuan Agriculture University, Chengdu 611130, China; xiaozq705@sina.com (Z.X.); tangshuyao705708@sina.com (S.T.); qiuling705@sina.com (L.Q.); 2 Institute of Ecological Agriculture, Sichuan Agricultural University, Chengdu 611130, China

**Keywords:** Triticeae, ND-FISH, chromosome, oligonucleotide probe, tandem repeats

## Abstract

Oligonucleotide probes and the non-denaturing fluorescence in situ hybridization (ND-FISH) technique are widely used to analyze plant chromosomes because they are convenient tools. New oligonucleotide probes, Oligo-Ku, Oligo-3B117.1, Oligo-3B117.2, Oligo-3B117.2.1, Oligo-3B117.3, Oligo-3B117.4, Oligo-3B117.5, Oligo-3B117.6, Oligo-pTa71A-1, Oligo-pTa71A-2, Oligo-pTa71B-1, Oligo-pTa71B-2, Oligo-pTa71C-1, Oligo-pTa71C-2, Oligo-pTa71C-3 and Oligo-pTa71D were designed based on the repetitive sequences KU.D15.15, pSc119.2-like sequence 3B117 and pTa71. Oligonucleotide probe (GT)_7_ was also used. Oligo-Ku and (GT)_7_ can be together used to identify *Dasypyrum villosum* from wheat chromosomes and to distinguish individual *D. villosum* chromosomes. The oligonucleotide probes that were derived from the same repeat sequence displayed different signal intensity and hybridization sites on the same chromosomes. Both the length and the nucleotide composition of oligonucleotide probes determined their signal intensity. For example, Oligo-3B117.2 (25 bp) and Oligo-pTa71A-2 (46 bp) produced the strongest signals on chromosomes of wheat (*Triticum aestivum* L.), rye (*Secale cereale* L.), barley (*Hordeum vulgare* ssp. *vulgare*) or *D. villosum*, the signal of Oligo-3B117.4 (18 bp) on the short arm of 7B chromosome was weaker than that of Oligo-3B117.2.1 (15 bp) and Oligo-3B117.3 (16 bp), and Oligo-pTa71A-1 (38 bp) produced the same strong signals as Oligo-pTa71A-2 did on 1B and 6B chromosomes, but its signals on 1R and 1V chromosomes were weaker than the ones of Oligo-pTa71A-2. Oligonucleotide probes and ND-FISH analysis can reflect the distribution and structural statues of different segments of tandem repeats on chromosomes. The possible reasons why different segments derived from the same repeat sequence produced different signal patterns are discussed.

## 1. Introduction

Since the non-denaturing fluorescence in situ hybridization (ND-FISH) technique was used to detect plant telomeres [1], it is often used to analyze plant chromosomes [2,3,4]. ND-FISH has also been widely used to study the chromosomes of wheat and its relatives [5,6,7,8,9,10,11,12,13]. A key step for successful ND-FISH analysis is to get suitable oligonucleotide probes. So far, two main types of oligonucleotide probes are often used for ND-FISH analysis of chromosomes of wheat and its relatives. They are simple sequence repeats (SSRs) [2,3,5,6,7] and non-SSR oligonucleotides [8,9,10,11,12,13]. Non-SSR oligonucleotide probes were designed according to the published tandem repeated sequences or transposable elements [8,9,12,13,14]. Some non-SSR oligonucleotide probes for ND-FISH analysis were obtained from high-throughput sequencing [9,13]. In fact, more new oligonucleotide probes can be developed from the known repetitive DNA sequences and be used for ND-FISH analysis of wheat and its relatives [13,14]. Tandem repeats pSc119.2 and pTa71 were often used to distinguish the chromosomes of wheat (*Triticum aestivum* L.), rye (*Secale cereale* L.), barley (*Hordeum vulgare* ssp. *vulgare*), *Dasypyrum villosum* and *Aegilops triuncialis* etc. [5,8,9,12,14,15,16,17]. In addition, some dispersed repetitive DNA sequences have been cloned from the genus *Secale* [18]. These known repetitive sequences provide rich resource for the developing new oligonucleotide probes. In the present study, these repetitive DNA sequences were used to develop some oligonucleotide probes that can produce new effects of ND-FISH analysis of wheat and its relatives.

## 2. Results

### 2.1. Signal Pattern of Different Segments of Tandem Repeat 3B117

**Figure 1:**
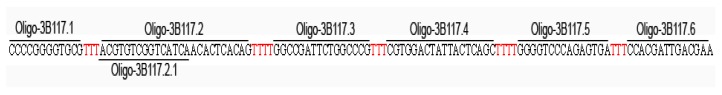
The nucleotide sequence of 3B117. The six segments that were used to design oligonucleotide probes were indicated.

**Figure 2:**
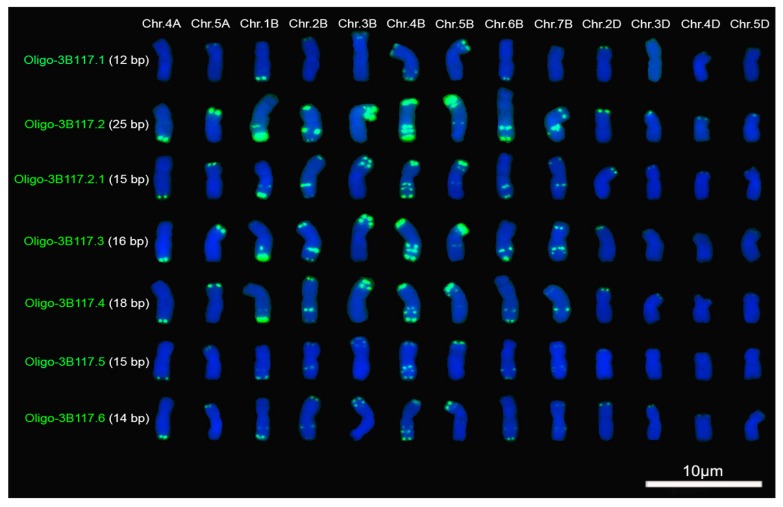
Signal patterns of the seven oligonucleotide probes (**green**) derived from 3B117 on the root tip metaphase chromosomes of common wheat Chinese Spring. Chr.: chromosome. Chromosomes were counterstained with 4′,6-diamidino-2-phenylindole (DAPI) (**blue**). Scale bar: 10 μm.

**Figure 3:**
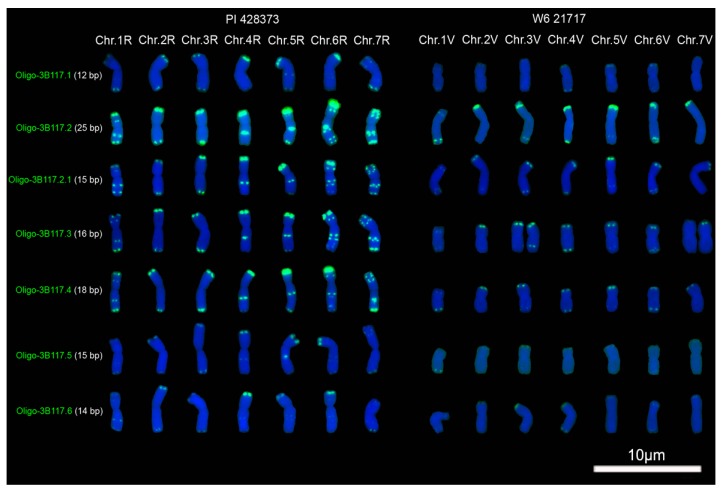
Signal patterns of the seven oligonucleotide probes (**green**) derived from 3B117 on the root tip metaphase chromosomes of rye PI 428373 and *D. villosum* W6 21717. Chr.: chromosome. Chromosomes were counterstained with 4′,6-diamidino-2-phenylindole (DAPI) (**blue**). Scale bar: 10 μm.

Tandem repeat 3B117 was derived from the sequence of the 3B chromosome of CS (IWGSC_RefSeq_V1_chr3B.fsa). 3B117 belongs to the 120-bp family of rye because it has 94% similarity with the pSc119.2 sequence. It can be noted that the sequence of 3B117 was divided into six segments by TTT (T) repeats (Figure 1). Therefore, the six segments were used to design seven oligonucleotide probes (Figure 1; Table 1). No signals of the seven oligonucleotide probes were observed on the barley chromosomes, and they produced different signal strength on the same chromosomes of CS, rye PI428373 and *D. villosum* W6 21717 (Figure 2 and Figure 3; Supplementary Materials Figures S1–S3). Oligo-3B117.2, which is the longest one among the seven probes, produced the strongest signals on the chromosomes of CS, rye PI428373 and *D. villosum* W6 21717 (Figure 2 and Figure 3; Figures S1–S3). The signals of Oligo-3B117.2.1, which contained the first 15 bases of Oligo-3B117.2, on the chromosomes of CS, rye PI428373 and *D. villosum* W6 21717 were weaker than that of Oligo-3B117.2 (Figure 2 and Figure 3; Figures S1–S3). This result indicates that the longer probe can produce stronger signal. However, this case is not going to happen all the time.

For CS, the signals of Oligo-3B117.1 (12 bp) on the telomeres of the long arm of 1B and the short arm of 2D chromosomes were stronger than that of Oligo-3B117.5 (15 bp) (Figure 2; Figure S1). The signal of Oligo-3B117.4 (18 bp) on the subtelomere of the short arm of 7B chromosome was weaker than that of Oligo-3B117.2.1 (15 bp) and Oligo-3B117.3 (16 bp) (Figure 2; Figure S1). The signals on 4A, 5A, 1B, 2B, 3B, 2D and 3D chromosomes produced by Oligo-3B117.6 (14 bp) were stronger than that produced by Oligo-3B117.5 (15 bp) (Figure 2; Figure S1). For rye PI428373, Oligo-3B117.1 (12 bp) and Oligo-3B117.6 (14 bp) produced stronger signals on the telomere of the short arms of 4R and 6R chromosomes than Oligo-3B117.5 (15 bp) did (Figure 3; Figure S2). There was no significant difference in the signal intensity between Oligo-3B117.4 (18 bp) and Oligo-3B117.2 (25 bp) on the telomere of the short arms of 5R and 6R chromosomes (Figure 3; Figure S2). For *D. villosum* W6 21717, the signal of Oligo3B117.3 (16 bp) on the telomere of the short arm of 3V chromosome was stronger than the one of Oligo-3B117.4 (18 bp) (Figure 3; Figure S3). The signal intensity of Oligo-3B117.2.1 and Oligo-3B117.5 was different, although they both have 15 bases (Figure 2 and Figure 3; Figures S1–S3).

In addition, the signals of Oligo-3B117.1 and Oligo-3B117.6 on the telomere of the short arm of 2B chromosome were stronger than that on the intercalary of the long arm of this chromosome, however, the opposite occurred for the other five probes (Figure 2; Figure S1). Oligo-3B117.1 only produced two signal bands on the long arm of 4B chromosome, and the other six probes produced three signal bands (Figure 2; Figure S1). From the centromere to the telomere, they can be successively named the first, the second and the third signal band.

For Oligo-3B117.5, the first signal band was stronger than the second one. However, for Oligo-3B117.4 and Oligo-3B117.6, the first signal bands were weaker than the second ones (Figure 2; Figure S1). No significant difference between the two signal bands of Oligo-3B117.2, Oligo-3B117.2.1 and Oligo-3B117.3 was observed (Figure 2; Figure S1). There were two signal bands on the intercalary of the long arm of 6R chromosome (Figure 3; Figure S2). From the centromere to the telomere, they can be named the first and the second signal band, successively. The first signal bands of Oligo-3B117.2, Oligo-3B117.2.1 and Oligo-3B117.4 were stronger than their second ones, and no great difference between the two signal bands of Oligo-3B117.3 was observed (Figure 3; Figure S2). The signal of Oligo-3B117.3 was missing from one of the long arms of the 3V chromosomes and was also absent from one of the 7V chromosomes (Figure 3; Figure S3). This result indicated that the 3V and 7V chromosomes in *D. villosum* W6 21717 were heterozygous.

### 2.2. Signal Pattern of Different Repeated Family of pTa71

**Figure 4:**
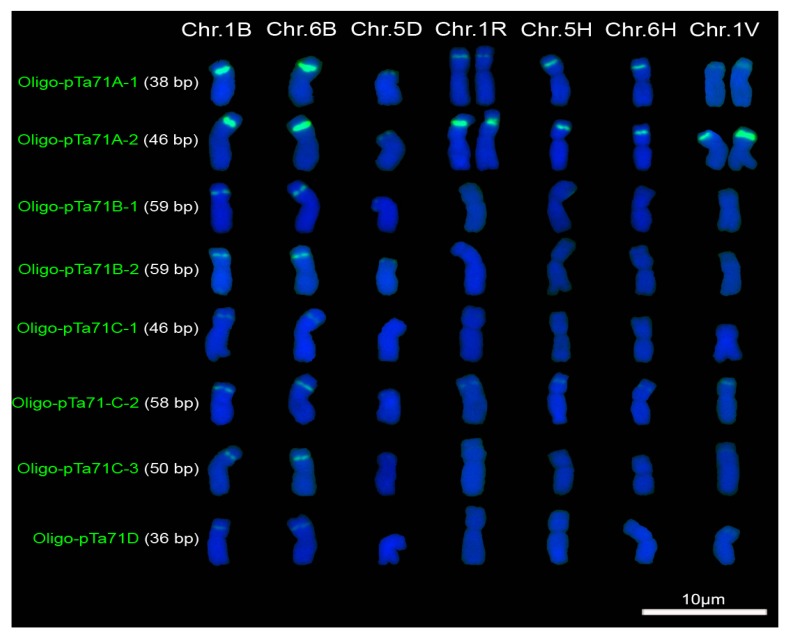
Signal patterns of the eight oligonucleotide probes (**green**) derived from pTa71 on the root tip metaphase chromosomes of common wheat Chinese Spring, rye PI 428373, barley cultivar CNSimai 1 and *D. villosum* W6 21717. Chr.: chromosome. Chromosomes were counterstained with 4′,6-diamidino-2-phenylindole (DAPI) (**blue**). Scale bar: 10 μm.

Oligo-pTa71A-1 produced very strong signals on 1B and 6B chromosomes, and its signals on 1R chromosomes were weak (Figure 4; Figures S4 and S5). The signal of Oligo-pTa71A-1 on 5D and 1V chromosomes, and the signals of Oligo-pTa71A-2, Oligo-pTa71B-1 and Oligo-pTa71B-2 on 5D chromosome were very weak (Figure 4; Figures S4, S5). The very strong signals of Oligo-pTa71A-2 on 1B, 6B, 1R and 1V chromosomes could be observed (Figure 4; Figures S4 and S5). On 5H and 6H chromosomes, the signals of both Oligo-pTa71A-1 and Oligo-pTa71A-2 were clear and strong (Figure 4; Figure S5).

Oligo-pTa71B-1, Oligo-pTa71B-2, Oligo-pTa71C-2 and Oligo-pTa71C-3 generated obvious signals on 1B and 6B chromosomes, although they were not very strong (Figure 4; Figure S4).Oligo-pTa71C-1 and Oligo-pTa71D produced weak signals on 1B and 6B chromosomes (Figure 4; Figure S4). Almost no signals of Oligo-pTa71C-1, Oligo-pTa71C-2, Oligo-pTa71C-3 and Oligo-pTa71D on 5D chromosome could be observed (Figure 4; Figure S4). Likewise, there were no signals of Oligo-pTa71B-1, Oligo-pTa71B-2, Oligo-pTa71C-1, Oligo-pTa71C-3 and Oligo-pTa71D on the 1R, 5H, 6H and 1V chromosomes (Figure 4; Figure S5). Oligo-pTa71C-2 produced very weak signals on 1R, 5H and 6H chromosomes, and its signal on 1V chromosome was weak (Figure 4; Figure S5). In addition, different signal intensity of Oligo-pTa71A-1 and Oligo-pTa71A-2 on the pair of 1R and the pair of 1V chromosomes was observed (Figure 4; Figure S5). This result indicats that the 1R chromosomes in rye PI 428373 and the 1V chromosomes in D. villosum W6 21717 were heterozygous.

### 2.3. ND-FISH Analysis for Identifying D. villosum Chromosomes in Wheat Backgrounds

**Figure 5:**
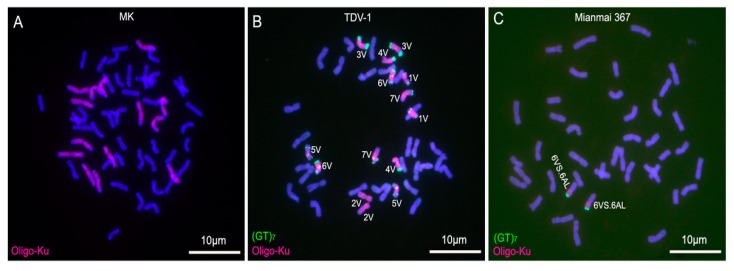
ND-FISH analysis using Oligo-Ku (**red**) and (GT)_7_ (**green**) as probes. (**A**) Root tip metaphase chromosomes of Octoploid triticale MK; (**B**) Root tip metaphase chromosomes of wheat–*D. villosum* amphiploid TDV-1; (**C**) Root tip metaphase chromosomes of common wheat cultivar Mianmai 367. Chromosomes were counterstained with 4′,6-diamidino-2-phenylindole (DAPI) (**blue**). Scale bar: 10 μm.

Octoploid triticale MK, *T. turgidum* cv. Jorc-69–*D. villosum* amphiploid TDV-1 and common wheat cultivar Mianmai 367 were analyzed by ND-FISH using oligonucleotide probes Oligo-Ku and (GT)_7_ (Table 1). Oligo-Ku produced hybridization signals on all 14 rye chromosomes in MK and on all 14 *D. villosum* chromosomes in TDV-1, but no signals were observed on the 42 wheat chromosomes (Figure 5). Probe (GT)_7_ produced signals on all 14 *D. villosum* chromosomes and no signals of (GT)_7_ were observed on the chromosomes of wheat and rye chromosomes (Figure 5). In addition, the seven *D. villosum* chromosomes can be distinguished by the signals of (GT)_7_ (Figure 5B). Probes Oligo-Ku and (GT)_7_ only produced signals on 6VS arms in wheat cultivar Mianmai 367 (Figure 5C). Therefore, Oligo-Ku and (GT)_7_ can be used together for ND-FISH assays to distinguish *D. villosum* chromosomes from wheat and rye chromosomes. 

## 3. Discussion

### 3.1. The Factors Influencing the Signal Intensity of Oligonucleotide Probes

Among the seven oligonucleotide probes that were derived from the repeated sequence 3B117, Oligo-3B117.2 was the longest probe and produced the strongest signals. The signals of Oligo-3B117.2.1, which contained partial bases of Oligo-3B117.2, were weaker than the ones of Oligo-3B117.2. This result indicates that longer probe produces stronger signal. It has already been reported that oligo probes with more nucleotides or more repeat units could produced stronger signals [13]. However, the results obtained in this study indicate that it isn’t always this way. For example, the signal of Oligo-3B117.4 (18 bp) on the subtelomere of the short arm of 7B chromosome was weaker than the ones of Oligo-3B117.2.1 (15 bp) and Oligo-3B117.3 (16 bp). Oligo-3B117.1 (12 bp), the shortest probe, produced stronger signal on the long arm of 1B chromosome and on the short arms of 4R and 6R chromosomes than Oligo-3B117.5 (15 bp) did (Figure 2 and Figure 3). In addition, the signals of Oligo-pTa71A-1 (38 bp) and Oligo-pTa71A-2 (46 bp) were stronger than that of Oligo-pTa71B-1 (59 bp), Oligo-pTa71B-2(59 bp), Oligo-pTa71C-1 (46 bp), Oligo-pTa71C-2 (58 bp) and Oligo-pTa71C-3 (50 bp) (Figure 4). In fact, the signal strength of Oligo-3B117.2 (25 bp) on wheat and rye chromosomes is not weaker than that of Oligo-pSc119.2-1 (45 bp) and Oligo-pSc119.2-2 (45 bp) reported by Fu et al. [9]. All these results indicate that the signal strength of oligonucleotide probes depends not only on the length of probes but also on the nucleotide composition. 

### 3.2. Oligonucleotide Probes and ND-FISH Reflecting Different Distribution Pattern of Tandem Repeats

In this study, the eight oligonucleotide probes that were named Oligo-pTa71 were designed according to the A, B, C and D repeat families that located within the wheat 25S-18S rDNA intergenic spacer [19]. Family A contains 12 direct repeats and the other three families only contain two or three direct repeats [19]. Therefore, it is easy to understand that the probe Oligo-pTa71A-2 could produce the strongest signals because it has the highest copy number among the four repeat families. However, how does one explain the variations of signal strength and signal sites of the seven oligonucleotide probes derived from 3B117. Likewise, how does one explain that Oligo-pTa71A-1 produced the same strong signals as Oligo-pTa71A-2 did on 1B and 6B chromosomes, but its signals on 1R and 1V chromosomes were weaker than the ones of Oligo-pTa71A-2. Two assumed reasons can be used to explain these questions. 

Firstly, distinct sequences derived from the same tandem repeat might target regions with different copy number on diverse chromosome regions. The diversity of pSc119.2 repeat family in tribe Triticeae has been studied [20]. The results indicated that nucleotide variation was distributed throughout the length of the repeat unit, no sequence homogenization occurred during the evolution of this repeat family and no characteristic genome or species-specific variants were observed [20]. That is, the variations of pSc119.2 repeat units are extensive. Therefore, different segments of 3B117 may target diverse chromosome regions each with a unique copy number. For example, the oligonucleotide probe Oligo-3B117.2 located on 4A, 5A and B-genome chromosomes with high copy number, and it produced very strong signals. Although oligonucleotide probe Oligo-3B117.2.1 was partial Oligo-3B117.2, it produced relatively weak signals because it was shorter and this was equivalent to decrease the number of copies. Oligo-3B117.5 produced weaker signals on 1B chromosome than Oligo-3B117.1 did because its copy number on 1B chromosome was lower than that of Oligo-3B117.1, even although it was longer than Oligo-3B117.1. Hence, it is logical to reason that the signal strength of oligonucleotide probes was determined not only by the length of probes but also by the nucleotide composition because different nucleotide composition had different copy number. If an oligonucleotide probe has high copy number, it can produce strong signal, even though it is short. So, oligonucleotide probes and ND-FISH analysis can reflect the distribution statues of different segments of tandem repeats on chromosomes.

Secondly, the different segments of the same tandem repeat might hybridize to distinctly different regions on chromosomes. It has already been reported that several oligonucleotide probes derived from the same repeated sequence showed different signal patterns and it was assumed that differently arranged or altered structural statuses of tandem repeats might exist on different chromosome regions [14]. A model of DNA repeat-assembled mitotic chromosomal skeleton has been reported [21]. In this model, tandem repeats formed tandem repeat assemblies (TRAs), which was the key component of chromoaxis [21]. Chromoaxis was a major structural element in the chromoskeleton model and it was buried in non-skeletal chromatin mass [21]. According to the chromoskeleton model, it can be assumed that some parts of a tandem repeat might be packaged tightly and its other parts were loosely structured in metaphase chromosomes. For example, it can be presumed that Oligo-pTa71A-1 segment was packaged tightly in the nucleolus organizer regions (NORs) of 1R and 1V chromosomes, the Oligo-pTa71A-2 segment was loosely structured, and both the Oligo-pTa71A-1 and Oligo-pTa71A-2 segments structured loosely in the NORs of 1B and 6B chromosomes. The capacity of the short oligonucleotide probes to invade chromosomal double-stranded DNA (dsDNA) was used to explain why ND-FISH analysis using SSRs as probes was successful, and it was suggested that strand invasion by oligonucleotides is a general phenomenon [2]. Therefore, it was difficult for probe Oligo-pTa71A-1 to invade chromosomal dsDNA in NORs of 1R and 1V chromosomes because it was tightly structured. Thus, oligonucleotide probes and ND-FISH analysis can reflect the structural statues of different segments of tandem repeats on chromosomes.

### 3.3. Convenience of Oligonucleotide Probes and ND-FISH in Identifying D. villosum Chromosomes in Wheat Backgrounds

*D. villosum* is a useful genetic resource in wheat breeding programs. The powdery mildew resistant gene *Pm21* on 6VS has already been used in commercial wheat cultivars [22]. During the utilization of *D. villosum* genetic material to improve wheat cultivars, it is essential to be able to distinguish and localize *D. villosum* chromatin in the wheat background. The genomic in situ hybridization (GISH) technique using *D. villosum* genomic DNA as a probe and the FISH technique using repetitive sequence as a probe have been often used to do this [17,22,23]. However, the routine procedures of GISH and FISH are time-consuming and labor-intensive. Therefore, ND-FISH technique using oligonucleotide probe Oligo-pHv62-1 was developed to conveniently identify *D. villosum* chromosomes in wheat background [12]. The signals of Oligo-pHv62-1 only presented in terminal or sub-terminal heterochromatic C-banding regions of *D. villosum* chromosomes [12], and this probe is difficult to identify the broken *D. villosum* chromosomes. In this study, oligonucleotide probe Oligo-Ku painted almost the entire *D. villosum* genome, therefore, its hybridization effect on *D. villosum* chromosomes is similar to the one of GISH. In addition, oligonucleotide probe (GT)_7_ not only could identify *D. villosum* from wheat chromosomes, but also could distinguish individual *D. villosum* chromosomes. Thus, Oligo-Ku and (GT)_7_ can be used together for ND-FISH analysis to conveniently identify *D. villosum* chromosomes in wheat background and distinguish the seven individual *D. villosum* chromosomes. It has been reported that oligonucleotide probes and the ND-FISH technique can replace genomic DNA of rye as probes to distinguish rye from wheat chromosomes, however, the oligonucleotide probes can’t distinguish individual rye chromosome [9]. Additionally, Oligo-Ku could also identify rye from wheat chromosomes and this provides additional oligonucleotide probe for identifying rye chromosomes.

## 4. Materials and Methods

### 4.1. Plant Materials

Common wheat Chinese Spring (CS), common wheat cultivar Mianmai 367, barley cultivar CNSimai 1, rye PI428373, *D. villosum* W6 21717, *T. turgidum* cv. Jorc-69–*D. villosum* amphiploid TDV-1 (genome AABBVV) [12] and octoploid triticale MK [9] were used in this study. Common wheat CS and octoploid triticale MK were provided by our laboratory. Mianmai 367 contained *D. villosum*–wheat 6VS.6AL translocation chromosomes [22] and was kindly provided by Dr. Yong Ren, Mianyang Academy of Agricultural Sciences, Sichuan, China. CNSimai 1 was kindly supplied by Professor Zongyun Feng, Agronomy College, Sichuan Agricultural University. Rye PI428373 was kindly provided by Professor Fangpu Han, Institute of Genetics and Developmental Biology, Chinese Academy of Science, Beijing, China. Amphiploid TDV-1 was friendly supplied by Professor Zujun Yang, School of Life Science and Technology, University of Electronic Science and Technology of China. *D. villosum* W6 21717 was from the American Germplasm Resources Information Network (GRIN).

### 4.2. Oligonucleotide Probe Development

The sequences of four repeat families that located in the intergenic region between the 25 S and 18 S wheat ribosomal RNA genes (GenBank accession number X07841.1) [19], the dispersed repetitive sequence KU.D15.15 (GenBank accession number GU318080.1) and the pSc119.2-like tandem repeat sequence 3B117 were used to design oligonucleotide probes. The sequences of four repeat families that located in the intergenic region between the 25 S and 18 S wheat ribosomal RNA genes were subcloned from the plasmid pTa71 [19]. Repetitive sequence KU.D15.15 belonging to the *Revolver* family was cloned from *S. cereale* Kustro [18]. Tandem repeat sequence 3B117 was derived from the sequence of 3B chromosome of CS, which was downloaded from International Wheat Genome Sequencing Consortium (IWGSC). The sequence 3B117 is displayed in Figure 1. 3B117 has 94% similarity with the pSc119.2 sequence [24], therefore, it belongs to the 120-bp family of rye. In addition, SSR probe (GT)_7_ was also used in this study. The names and the sequences of the oligonucleotide probes used in this study are listed in Table 1.

### 4.3. ND-FISH Analysis

The oligonucleotide probes listed in Table 1 were used for ND-FISH analysis. Oligonucleotide probes were synthesized by Tsingke Biological Technology Co. Ltd. (Beijing, China). The oligonucleotide probe Oligo-Ku was 5′–end-labelled with 6-carboxytetramethylrhodamine (TAMRA) (Table 1). The other oligonucleotide probes were 5′–end-labelled with 6-carboxyfluorescein (6-FAM) (Table 1). The chromosome spreads of materials were prepared through the methods described by Han et al. [25]. The synthesized oligonucleotide probes were diluted by using 1× TE solution (pH 7.0). Probe amounts per slide are listed in Table 1. The probe mixture containing each probe, 2× SSC and 1× TE buffer (pH 7.0, total volume = 10 µL) was dropped at the center of the cell spreads, and covered with glass coverslip. When dropped the probe mixture at the cell spreads, the room temperature was kept between 25 °C and 28 °C. Slides were immediately stored in a moist box at 42 °C for 1 h and washed 15 s in 2 × SSC with the temperature 42 °C. In addition, (AAG)_6_, (AAC)_6_, Oligo-pSc119.2-1 and Oligo-pTa535-1 [8] were also used to help identify wheat, rye and *D. villosum* chromosomes. Probes (AAG)_6_, (AAC)_6_ and Oligo-pSc119.2-1 were 5′–end-labeled with TAMRA. Oligo-pTa535-1 was 5′–end-labeled with Cyanine Dye 5 (Cy5). For each newly developed oligonucleotide probe (Table 1), ND-FISH was repeated three times. At least five metaphase cells were examined for each slide.

An epifluorescence microscope (BX51, Olympus) equipped with a cooled charge-coupled device camera operated with HCIMAGE Live software (version 2.0.1.5, Hamamatsu Corporation, Sewickley, NJ, USA) was used to take images. For probes Oligo-3B117.1, Oligo-3B117.2, Oligo-3B117.2.1, Oligo-3B117.3, Oligo-3B117.4, Oligo-3B117.5, Oligo-3B117.6, Oligo-pTa71A-1, Oligo-pTa71A-2, Oligo-pTa71B-1, Oligo-pTa71B-2, Oligo-pTa71C-1, Oligo-pTa71C-2, Oligo-pTa71C-3 and Oligo-pTa71D, the exposure time was 150 ms. The exposure time of probe Oligo-Ku and (GT)_7_ was 200 ms. 

## 5. Conclusions

In conclusion, new oligonucleotide probes have been developed according to the published repetitive DNA sequences. Oligo-Ku and (GT)_7_ can be used to conveniently identify *D. villosum* from wheat chromosomes and to distinguish individual *D. villosum* chromosomes. Oligo-3B117.2 and Oligo-pTa71A-2 are recommended to be used to identify chromosomes of wheat, rye, barley and *D. villosum* because they can produce very strong signals on these chromosomes. The oligonucleotide probes that were derived from the same repeat sequence displayed different signal intensity and hybridization sites on the same chromosomes. The signal strength of oligonucleotide probes depends not only on the length of probes but also on the nucleotide composition. Oligonucleotide probes and ND-FISH analysis can reflect the distribution and structural statues of different segments of tandem repeats on chromosomes. The results obtained in this study provide a reference for developing new oligonucleotide probes for ND-FISH analysis of wheat and its relatives.

## Figures and Tables

**Table 1:** Oligonucleotide probes for ND-FISH analysis.

Probe	Nucleotide Sequence and Fluorochrome Label	Length of Probe (bp)	Amount Used for Each Slide (pmol/slide)	Original Sequences Used to Develop Probes (GenBank Accession Number)
Oligo-Ku	Tamra-5′GATCG AGACT TCTAG CAATA GGCAA AAATA GTAAT GGTAT CCGGG TTCG 3′	49	0.68	Rye dispersed repetitive sequence KU.D15.15 (GU318080.1)
Oligo-3B117.1	6-FAM-5′CCCCGGGGTGCG3′	12	2.0	3B117, obtained from sequence of IWGSC_RefSeq_V1_chr3B.fsa
Oligo-3B117.2	6-FAM-5′ACGTGTCGGTCATCAACACTCACAG3′	25	2.0	
Oligo-3B117.2.1	6-FAM-5′ACGTGTCGGTCATCA3′	15	2.0	
Oligo-3B117.3	6-FAM-5′GGCCGATTCTGGCCCG3′	16	2.0	
Oligo-3B117.4	6-FAM-5′CGTGGACTATTACTCAGC3′	18	2.0	
Oligo-3B117.5	6-FAM-5′GGGGTCCCAGAGTGA3′	15	2.0	
Oligo-3B117.6	6-FAM-5′CCACGATTGACGAA3′	14	2.0	
Oligo-pTa71A-1	6-FAM-5′CCGTG AACGG GCTGT ACGAG GACAC GGGAA AAAAC TGG3′	38	1.0	Wheat ribosomal DNA (rDNA) 25S-18S intergenic region *EcoR*I-*BamH*I fragment, repeat family A (X07841.1)
Oligo-pTa71A-2	6-FAM-5′CCGAC GGCCG TCGTG GACGG AAGTT GACGC GCGCC ATGGA AAACT G3′	46	1.0	
Oligo-pTa71B-1	6-FAM-5′AAATG GCTAA GTCCC TTGTA AGACA TACCC TTGGA CCCAA TAAGG CCAGT GGAAA CAAC3′	59	1.0	Wheat ribosomal DNA (rDNA) 25S-18S intergenic region *EcoR*I-*BamH*I fragment, repeat family B (X07841.1)
Oligo-pTa71B-2	6-FAM-5′TACTT GGCCG ATTCA TGCGG ATGCC GTCGT CAGAG GCTAC ACTGC TAAGT CATGG TCAA3′	59	1.0	
Oligo-pTa71C-1	6-FAM-5′GTCGC CTCCG GAAAA ACGTT GCCCC TCGGT GGCAA CGTCA TCGCT GT3′	46	1.0	Wheat ribosomal DNA (rDNA) 25S-18S intergenic region *EcoR*I-*BamH*I fragment, repeat family C (X07841.1)
Oligo-pTa71C-2	6-FAM-5′TGTAC GTCTC AAGTG AAATT CTAAC CCAAC AGCCG AATGC GGCTC GGGAA ACAGG AAA3′	58	1.0	
Oligo-pTa71C-3	6-FAM-5′CCCGT TGCGT ACACG ATCCG ACCGA CGGTA AACAG TCGCA ACGAT GTCCC3′	50	1.0	
Oligo-pTa71D	6-FAM-5′CATGT CTCAT GGCAA AAAAA CGCTG CCACG GCAGC G 3′	36	1.0	Wheat ribosomal DNA (rDNA) 25S-18S intergenic region *EcoR*I-*BamH*I fragment, repeat family D (X07841.1)
(GT)_7_	6-FAM-5′GTGTGTGTGTGTGT3′	14	1.77	

## Supplementary Materials

The following are available online.
