# Supplementary figures and images for: Oligonucleotides and ND-FISH Displaying Different Arrangements of Tandem Repeats and Identification of Dasypyrum villosum Chromosomes in Wheat Backgrounds
Source: Molecules. 2017 Jun 14;22(6):973. doi: 10.3390/molecules22060973 (PMC6152725; doi:10.3390/molecules22060973)

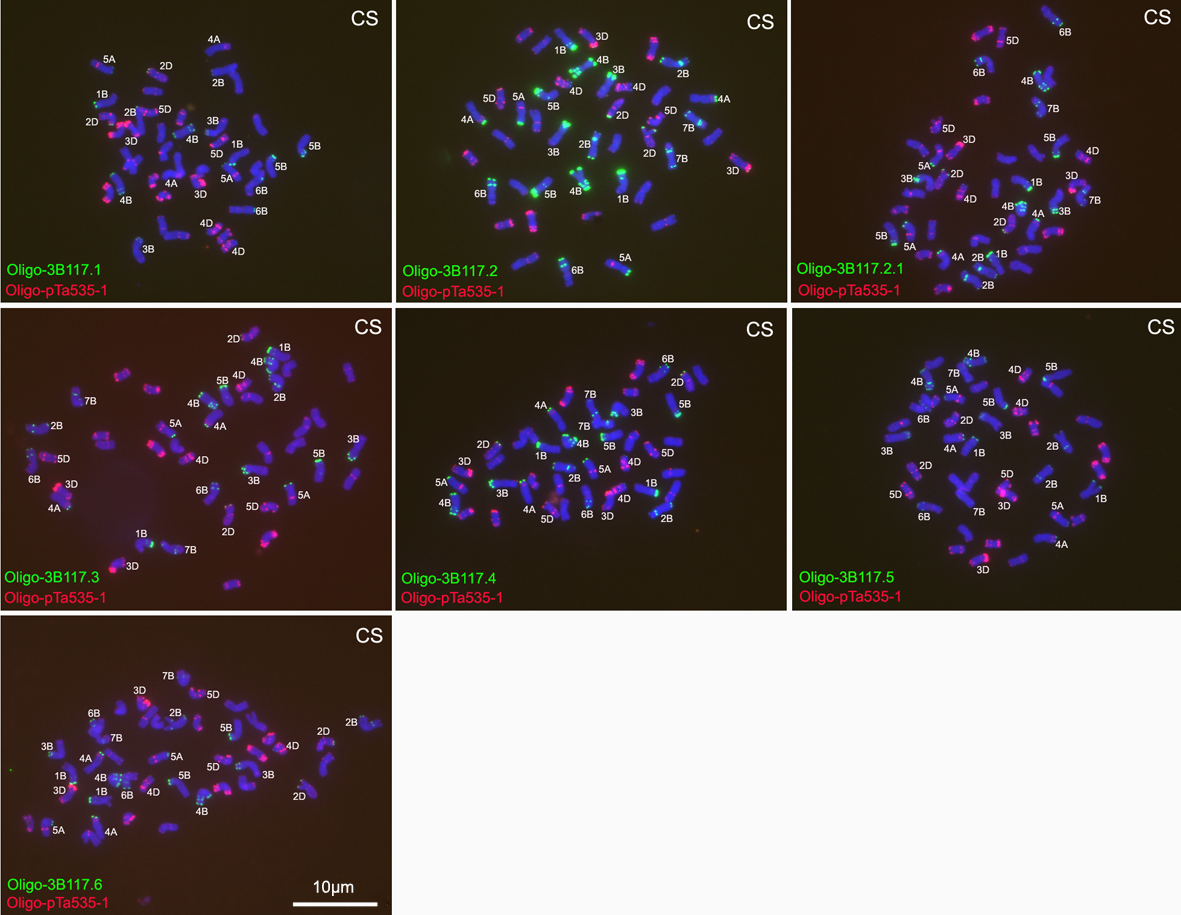

Supplement: Supplementary file 1 [file molecules-22-00973-s001.zip › Revised-Figure S1-S5/Figure S1.tif]

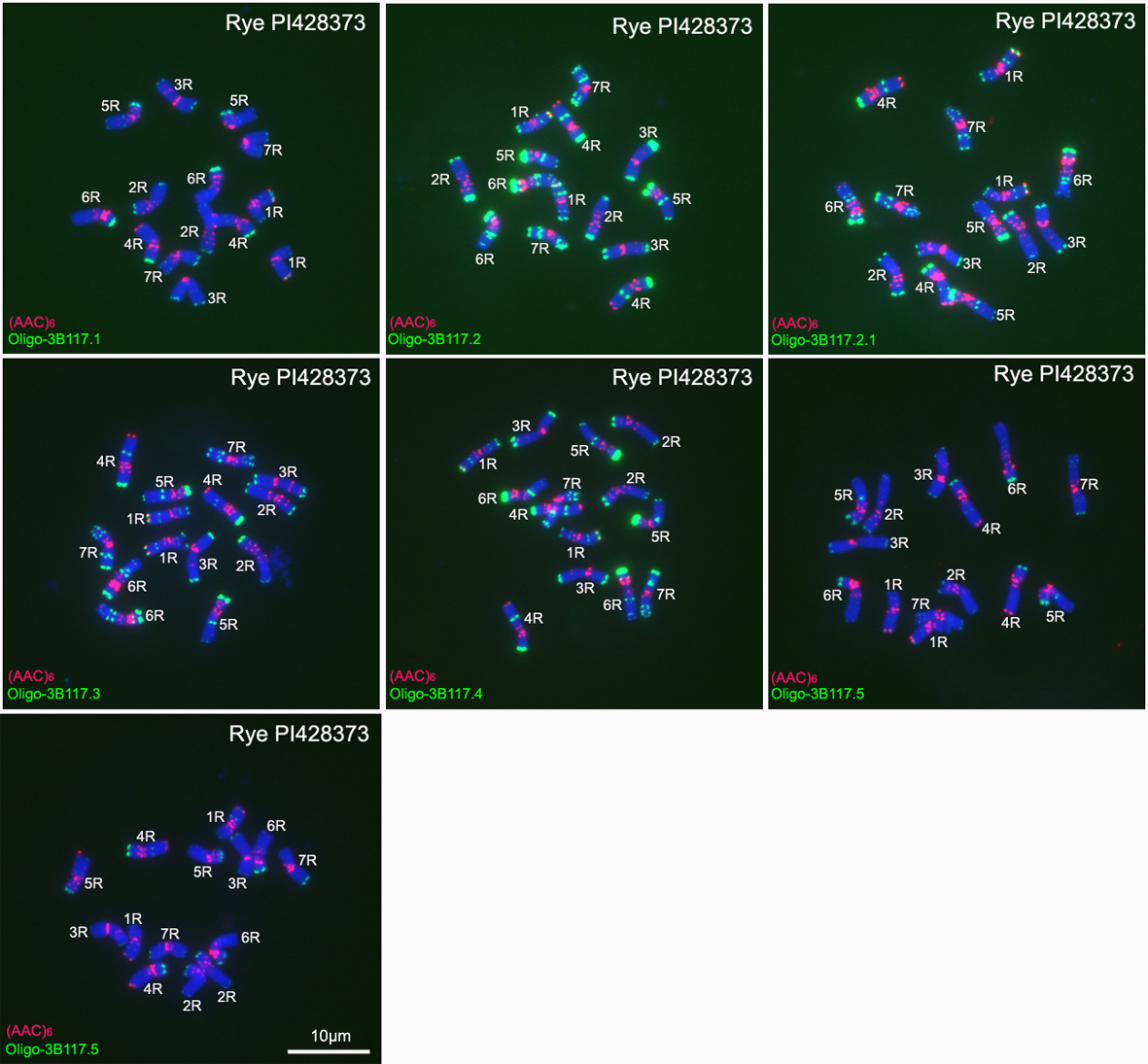

Supplement: Supplementary file 1 [file molecules-22-00973-s001.zip › Revised-Figure S1-S5/Figure S2.tif]

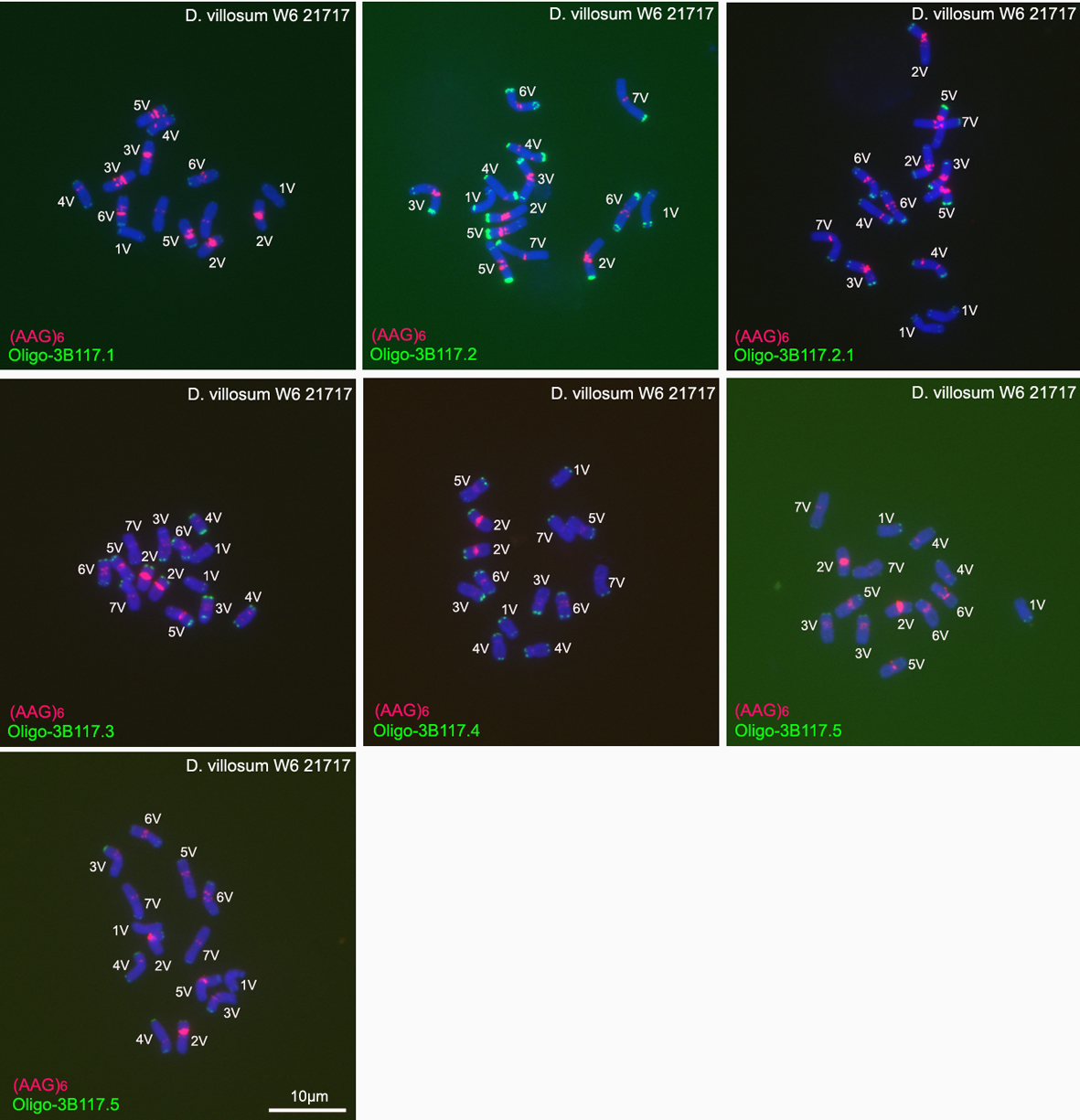

Supplement: Supplementary file 1 [file molecules-22-00973-s001.zip › Revised-Figure S1-S5/Figure S3.tif]

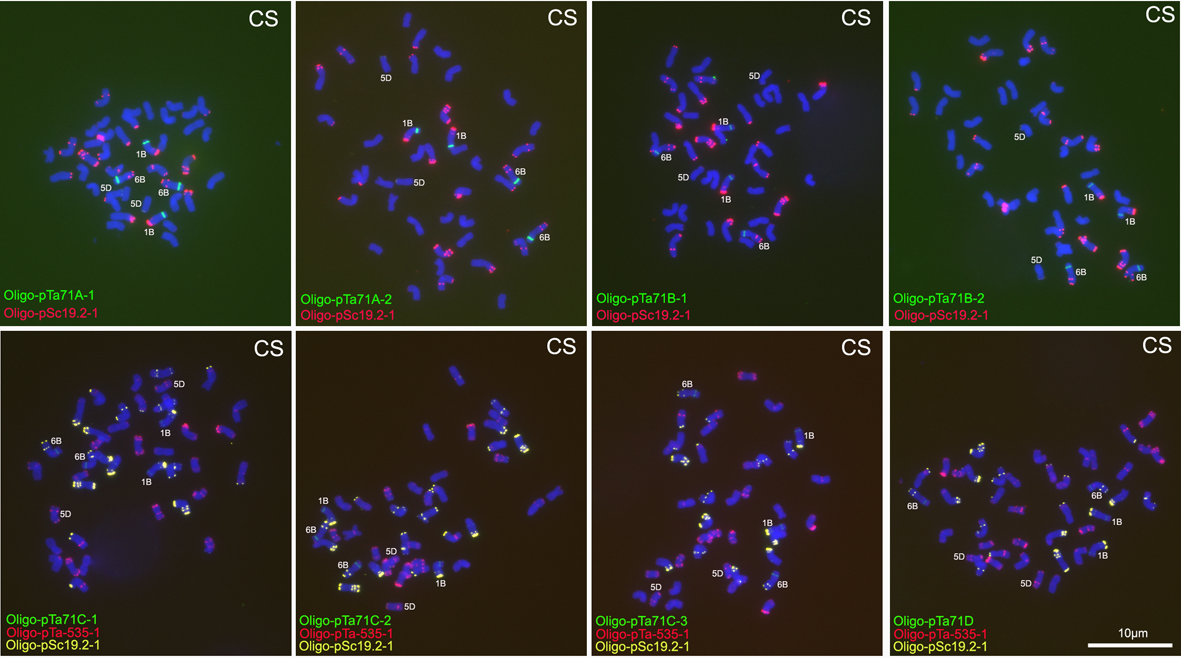

Supplement: Supplementary file 1 [file molecules-22-00973-s001.zip › Revised-Figure S1-S5/Figure S4.tif]

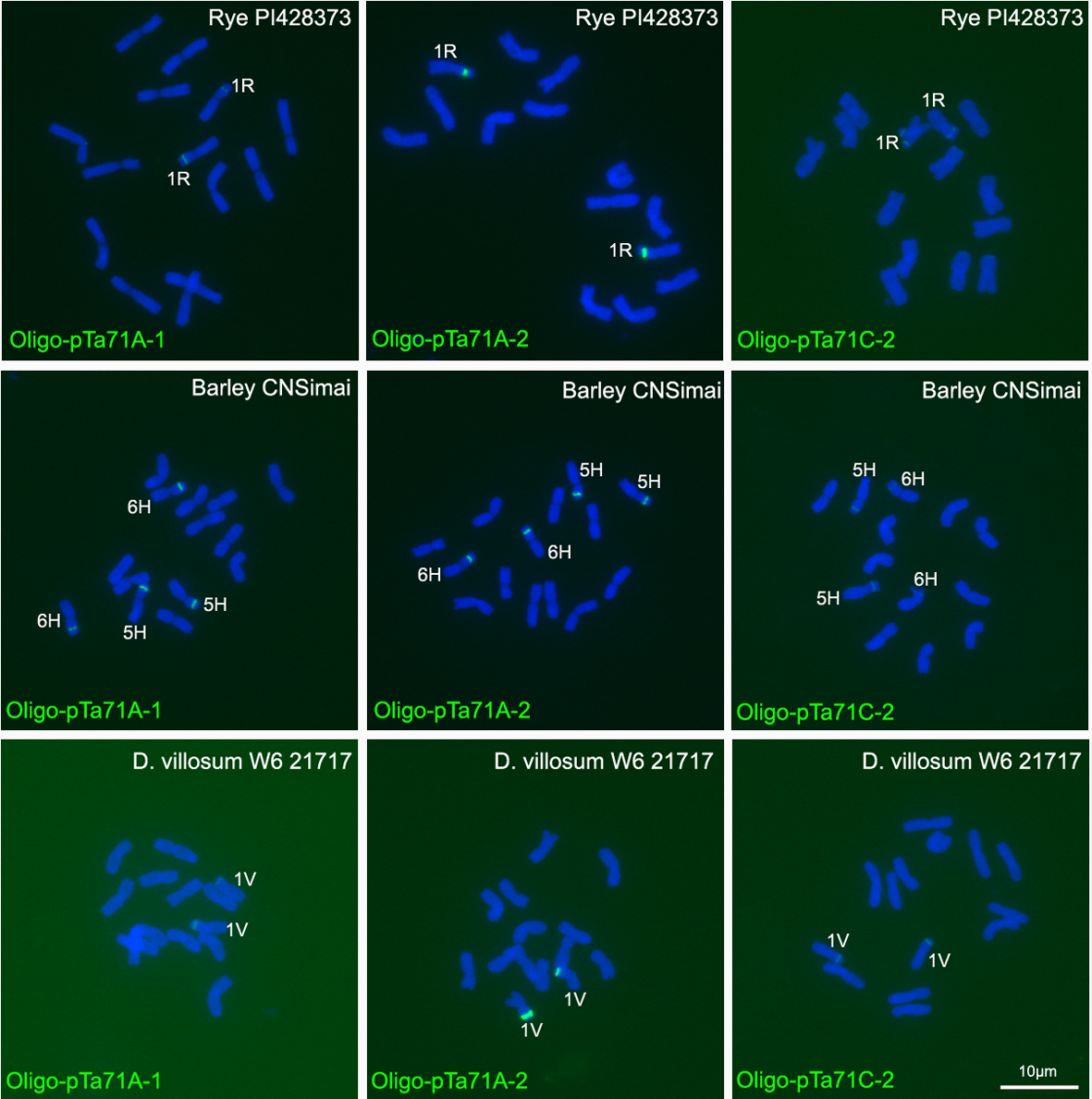

Supplement: Supplementary file 1 [file molecules-22-00973-s001.zip › Revised-Figure S1-S5/Figure S5.tif]
